# Diagnostic Yield of Cardiac CT to Detect Cardiac Thrombi in Patients With Acute Ischemic Stroke (AIS of HEARTS)

**DOI:** 10.1161/STROKEAHA.126.055575

**Published:** 2026-06-02

**Authors:** Shan Sui Nio, Daniel S. Green, Alexander Berry-Noronha, Leon A. Rinkel, Chiel F.P. Beemsterboer, Md Golam Hasnain, R. Nils Planken, Adrienne van Randen, Sinan Al-Hadethi, Mirre G.M. Hilt, Deborah S.A. Ruhe, Berto J. Bouma, S. Matthijs Boekholdt, Beng Lim Alvin Chew, Joshua Fridgant, Luis Mena-Romo, Dong Hyun Kim, Shuyu Guo, Yassar Alamri, Joel Winders, Sajith C. Senadeera, Anthony T. Lim, John N. Fink, Melissa Leung, Dennis Cordato, Mark W. Parsons, Neil J. Spratt, Carlos Garcia-Esperon, Teddy Y. Wu, Jonathan M. Coutinho

**Affiliations:** 1 Department of Neurology (S.S.N., L.A.R., C.F.P.B., M.G.M.H., D.S.A.R., J.M.C.), Amsterdam UMC, location University of Amsterdam, Meibergdreef 9, the Netherlands.; 2 Department of Radiology (R.N.P., A.v.R.), Amsterdam UMC, location University of Amsterdam, Meibergdreef 9, the Netherlands.; 3 Department of Cardiology (B.J.B., S.M.B.), Amsterdam UMC, location University of Amsterdam, Meibergdreef 9, the Netherlands.; 4 Department of Neurology (D.S.G., D.C., M.W.P.), School of Clinical Medicine, South Western Sydney Clinical Campus, University of New South Wales, Australia.; 5 Department of Cardiology (M.L.), School of Clinical Medicine, South Western Sydney Clinical Campus, University of New South Wales, Australia.; 6 Ingham Institute for Applied Medical Research, Liverpool, New South Wales, Australia (D.S.G., M.L., D.C., M.W.P.).; 7 Department of Neurology (A.B.-N., D.H.K., S.G., J.W., J.N.F., T.Y.W.), Christchurch Hospital, New Zealand.; 8 Department of Radiology (S.C.S., A.T.L.), Christchurch Hospital, New Zealand.; 9 Department of Neurology, School of Medicine and Public Health, University of Newcastle, Australia (M.G.H.).; 10 Department of Radiology, Mayo Clinic, Rochester, MN (R.N.P.).; 11 Department of Radiology, John Hunter Hospital, Newcastle, New South Wales, Australia (S.A.-H.).; 12 Department of Neurology, John Hunter Hospital, College of Health, Medicine and Wellbeing, University of Newcastle, Australia (B.L.A.C., J.F., L.M.-R., N.J.S., C.G.-E.).; 13 Department of Medicine, University of Otago, Christchurch, New Zealand (Y.A., T.Y.W.).; 14 Department of Cardiology (M.L.), Liverpool Hospital, New South Wales, Australia.; 15 Department of Neurology (D.C., M.W.P.), Liverpool Hospital, New South Wales, Australia.

**Keywords:** acute ischemic stroke, cardiac thrombus, cardioembolic, embolism, heart, tomography

## Abstract

**BACKGROUND:**

Cardiac computed tomography (CT) acquired during the acute stroke imaging protocol is an emerging modality to detect cardiac thrombi. We determined its yield in patients with acute ischemic stroke.

**METHODS:**

We performed a 1-stage individual patient data meta-analysis of 4 prospective observational cohorts (AIS of HEARTS [Acute Ischemic Stroke of Heart-Related Embolic Sources Detected on Acute Cardiac CT Scans]), including patients with acute ischemic stroke who underwent ECG-gated or non–ECG-gated cardiac CT between May 2018 and June 2024. We excluded patients with transient ischemic attack or stroke mimics. The primary outcome was the proportion of patients with a thrombus on cardiac CT. Secondary outcomes were additional scan time, radiation dose, comparison with echocardiography, and 90-day outcomes. We performed logistic regression analyses to compare 90-day outcomes between patients with and without thrombus, adjusting for age, sex, history of atrial fibrillation, ischemic heart disease, chronic heart failure, stroke or transient ischemic attack, anticoagulant use, prestroke modified Rankin Scale score, National Institutes of Health Stroke Scale score, large vessel occlusions, and intravenous thrombolysis, as appropriate for each outcome.

**RESULTS:**

We included 3919 patients (median age, 74 [interquartile range (IQR), 63–82], 58% male, median National Institutes of Health Stroke Scale score 6 [IQR, 3–12]). Cardiac CT detected a thrombus in 243 (6.2%) patients. Among 1323 patients that underwent both cardiac CT and transthoracic echocardiography, cardiac CT had a higher yield than transthoracic echocardiography (odds ratio, 7.4 [95% CI, 4.0–15.1]; *P*<0.001). Median additional scan time was 6 minutes (IQR, 5–7) for ECG-gated and 13 seconds (IQR, 12–61) for non–ECG-gated cardiac CT. Median additional radiation dose was 2.9 mSv (IQR, 1.6–4.1). Patients with thrombi had higher 90-day mortality (33% versus 15%, adjusted odds ratio, 1.6 [95% CI, 1.1–2.3]) and worse modified Rankin Scale scores (median modified Rankin Scale score 3 versus 2, adjusted odds ratio, 1.6 [95% CI, 1.2–2.0]), but similar recurrent stroke rates (5% versus 4%, adjusted odds ratio, 1.4 [95% CI, 0.7–2.5]).

**CONCLUSIONS:**

Implementing cardiac CT into the acute stroke imaging protocol is feasible, detects thrombi in ≈6% of patients, and has a higher yield than transthoracic echocardiography. Cardiac thrombi were associated with higher mortality, but not higher stroke recurrence.

**REGISTRATION:**

URL: https://www.clinicaltrials.gov; Unique identifier: NCT07165093.

Acute ischemic stroke (AIS) is one of the leading causes of death and disability worldwide.^1^ Moreover, up to 20% of patients experience a recurrent stroke within 5 years.^2^ Detecting the source of AIS allows targeted stroke prevention strategies, but with current diagnostic modalities, the rate of cryptogenic stroke still lies around 25%.^3^ It is believed that part of cryptogenic strokes are due to an undetected cardioembolic source.^3,4^ Patients with cardioembolic stroke generally suffer more severe strokes, have a higher risk of stroke recurrence, and require treatment with anticoagulants rather than antiplatelet therapy.^4^

Transthoracic echocardiography (TTE) is the most commonly used modality to detect structural cardioembolic sources, such as intracardiac thrombi.^5^ Visualizing the left atrial appendage (LAA), which is the most frequent location of cardiac thrombi in AIS, however, is difficult with TTE.^6^ Furthermore, TTE is often performed days or weeks after the AIS, and according to an international survey among neurologists, TTE is not performed as part of the standard stroke workup in ≈30% of cases.^5^ The same survey also showed that transesophageal echocardiography (TEE) is rarely performed as part of the routine workup despite its higher diagnostic yield compared with TTE, most likely due to its invasiveness, burden on the patient, and small risk of complications.^5,7^

Cardiac computed tomography (CT) acquired during the acute stroke imaging protocol is an emerging diagnostic modality to detect cardiac thrombi in patients with AIS. Studies have shown a higher diagnostic yield of cardiac CT to detect cardiac thrombi than TTE, but these studies mostly had modest sample sizes.^8–10^

We investigated the diagnostic yield of cardiac CT acquired during the acute stroke scan protocol to detect cardiac thrombi in patients with AIS, using individual patient data from a large international observational study.

## Methods

The data will be made available on reasonable request to the corresponding author.

### Study Design and Patients

We performed an individual patient data meta-analysis, including patients from 4 prospective observational cohorts: Christchurch Hospital, New Zealand (inclusion September 2018–June 2024), Amsterdam University Medical Center (UMC), the Netherlands (May 2018–June 2024), John Hunter Hospital, Australia (November 2020–June 2024) and Liverpool Hospital, Australia (October 2022–June 2024). These centers founded the AIS of HEARTS collaboration (Acute Ischemic Stroke of Heart-Related Embolic Sources Detected on Acute CaRdiac CT Scans). The aims of the collaboration and steering committee are listed in Table S1. For all centers, research was approved by the local medical ethics research committee (reference numbers: Amsterdam-018_017#C2018275; John Hunter Hospital and Liverpool-Hunter New England Local Health District Human Research Ethics Committee 11/08/17/4.01, Christchurch–National Health Disability Committee 17/CEN/84) and conducted in accordance with the applicable national guidelines. Patients or legal representatives of Amsterdam UMC provided written consent. In case of no written consent, opt-out consent was used. For John Hunter and Liverpool Hospital, opt-out consent was used. Christchurch Hospital waived written consent. This study was reported according to the MOOSE guidelines (Meta-Analyses of Observational Studies in Epidemiology).^11^

For data harmonization between centers, we developed a shared data dictionary that specified each variable to be collected from the 4 participating centers. The pooled data set was curated centrally, and regular meetings were held between investigators from all participating centers to review data quality and monitor data completeness.

Cardiac CT was performed as part of the acute stroke scan protocol in adult patients with acute stroke-like symptoms who were potentially eligible for reperfusion treatment (stroke onset <24 hours). We excluded patients with a diagnosis other than AIS (ie, transient ischemic attack [TIA] or stroke mimic). A list of variable definitions is shown in Table S2.

### Scan Acquisition and Diagnostic Procedures

The acute stroke scan protocol at each hospital started with a noncontrast brain CT, followed by a CT perfusion and a CT angiography (CTA) of head and neck vessels. At Christchurch Hospital, all patients underwent a non–ECG-gated cardiac CT as an extension of the CTA of the intracranial and carotid vessels with the scan window extended to include the LAA. A second delayed cardiac CT was obtained 30 seconds after the start of the CTA contrast bolus. At John Hunter Hospital, all patients underwent a non–ECG-gated cardiac CT, which was obtained 30 seconds and again 2 minutes after the start of the CTA contrast bolus. At Amsterdam UMC and Liverpool Hospital, patients underwent a prospective ECG-gated cardiac CT after the CTA. Further details regarding scan protocols and hospital characteristics are provided in Tables S3 and S4 and Figure S1.

The assessment of cardiac CT was initially performed by the local on-call radiologist, as this was part of the acute stroke scan protocol. All cardiac CT scans were assessed for a second time by a neurologist, a PhD student with extensive experience in reading cardiac CT scans, or a cardiac imaging cardiologist. A cardiac radiologist or cardiac imaging cardiologist reviewed all cardiac CT scans with a suspected cardiac thrombus or filling defect that was difficult to distinguish from slow-flow, according to the local definitions (Table S5). They were not blinded to clinical data for the cardiac CT assessments.

As part of the standard stroke workup, each patient received a 12-lead ECG, and patients admitted to the stroke unit additionally underwent at least 24 hours of rhythm monitoring, except at Christchurch Hospital. Outpatient Holter monitoring was not routinely performed in all patients. TTE was performed in a selection of patients after cardiac CT, depending on clinical features, availability, and local logistics. TEE was only performed in special situations where it was deemed required by the attending cardiologist and neurologist. Local cardiologists assessed the echocardiographic examinations, without blinding to the cardiac CT results.

### Clinical Data Acquisition

Follow-up at 90 days was performed as part of routine care, either by structured telephone interview or outpatient clinic visit. We measured functional outcome with the modified Rankin Scale (mRS; score 0=no symptoms, 6=death). In addition, we collected data on clinical events: recurrent ischemic stroke, TIA, intracerebral hemorrhage, major bleeding, cardiac events, systemic arterial occlusion, and venous thromboembolism. Major adverse cardioembolic events (MACE) were defined as a composite of nonfatal ischemic stroke, nonfatal myocardial infarction, and cardiovascular death. In case patients could not be reached, follow-up data were obtained from general practitioners, other treating hospitals, or retrieved from medical files.

### Outcomes

The primary outcome was the proportion of patients with a cardiac thrombus detected on acute cardiac CT. Cardiac thrombi could be located in the LAA, left atrium (LA), or left ventricle (LV).

As a secondary outcome, we compared the proportion of patients with a cardiac thrombus detected on acute cardiac CT versus echocardiography, among patients who underwent both modalities. In addition, we investigated the additional radiation dose by multiplying the cardiac CT dose-length product (DLP) by a conversion factor *k* of 0.014 mSv·mGy^−1^·cm^−^^1^ for cardiac CT.^12^ We also calculated additional scan time of the cardiac CT (defined as time from end of CTA until end of cardiac CT). Clinical outcomes at 90 days were mRS score, mortality, recurrent ischemic stroke, and MACE. We also assessed the yield of cardiac CT across the following subgroups: age (split across the median), sex, atrial fibrillation (AF) at baseline, large vessel occlusion (LVO), onset-to-hospital door time (split across median), and National Institutes of Health Stroke Scale (NIHSS) score (NIHSS score <5, NIHSS score 5–10, NIHSS score >10).

### Statistical Analyses

We report the proportion of patients with a cardiac thrombus. We used a McNemar test to compare the diagnostic yield of cardiac CT with TTE and TEE separately and report the odds ratio (OR) with 95% CI and the mid-*P* value. We used an ordinal logistic regression model to calculate the difference in functional outcome and a binomial logistic regression model to calculate the difference in mortality, recurrent ischemic stroke, and MACE between patients with and without a cardiac thrombus. We adjusted for the following potential confounders in the model for functional outcome and mortality: age, sex, history of AF, ischemic heart disease and chronic heart failure, anticoagulant use, prestroke mRS score, NIHSS score, LVO, and intravenous thrombolysis treatment. In the model for recurrent ischemic stroke, we adjusted for age, sex, history of AF and ischemic stroke or TIA and anticoagulant use, and for MACE: age, sex, history of AF, ischemic stroke or TIA, ischemic heart disease, and chronic heart failure, anticoagulation use, and NIHSS score. We report the unadjusted and adjusted OR. We performed a sensitivity analysis, including hospital location as a random effect in the outcome regression analyses, to account for potential differences between hospitals. For the subgroup analysis, we used a χ^2^ test or a Fisher exact test as appropriate. We report the percentage of detected cardiac thrombi and 95% CI. For all tests, we used a 2-sided significance level of 0.05. Analyses were performed using R version 4.4.2 (R Foundation for Statistical Computing 2024).

## Results

Of 6392 patients who presented with possible AIS and who underwent acute cardiac CT, we excluded 2473 patients, mostly because patients were diagnosed with a TIA (n=369) or stroke mimic (n=1815; Figure S2). Therefore, the study population consisted of 3919 patients with AIS.

The median age was 74 (interquartile range [IQR], 63–82), and 2273 (58%) were male patients (Table 1; Table S6). Common baseline cardiovascular risk factors were previous ischemic stroke or TIA (25%), AF (23%), and hypertension (57%). The median baseline NIHSS score was 6 (IQR, 3-12), and 1292 (33%) patients had an LVO. A total of 1804/3912 (46%) received reperfusion therapy: 863 intravenous thrombolysis, 594 endovascular treatment, and 347 both. In patients with known stroke onset, time from stroke to cardiac CT was 140 minutes (IQR, 97–253).

**Table 1.**
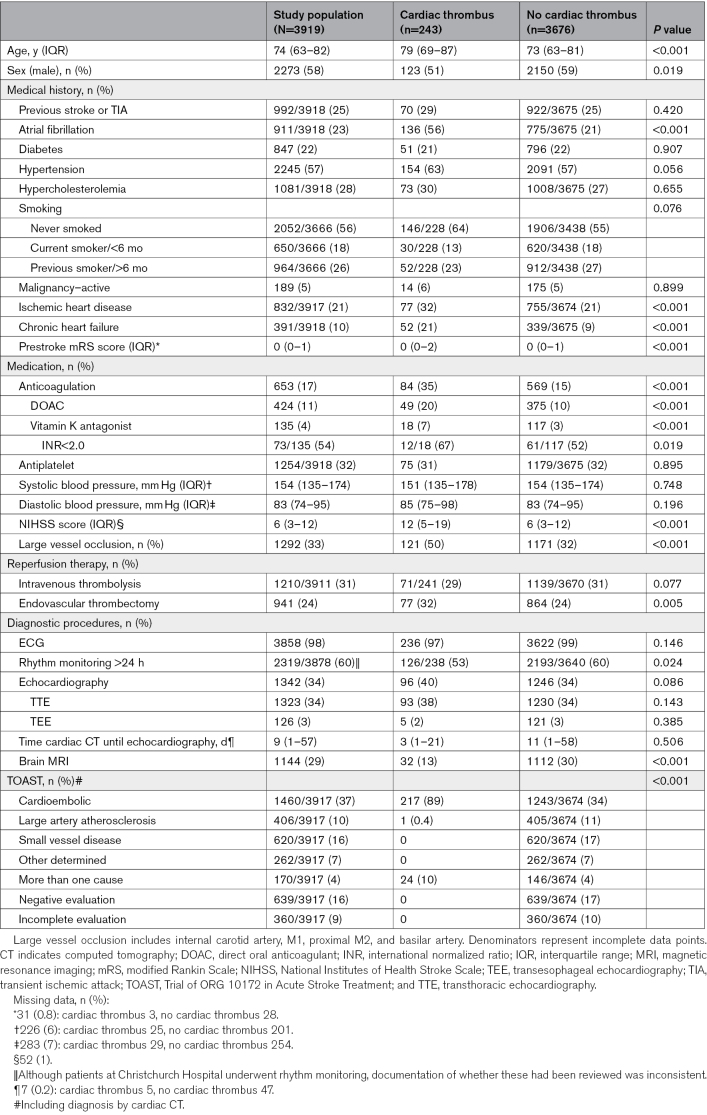
Baseline Characteristics Study Population and Stratified by the Presence of Cardiac Thrombus

In total, 1457 (37%) patients underwent ECG-gated cardiac CT, and 2462 (63%) non–ECG-gated cardiac CT (Table S7). ECG-gated cardiac CT extended the scan protocol by a median of 6 (IQR, 5–7) minutes. For non–ECG-gated cardiac CT, this was 13 (12–61) seconds. Actual scan time for ECG-gated cardiac CT was 4 seconds. The median additional radiation dose was 2.9 mSv (IQR, 1.6–4.1) for the total population, 2.0 mSv (1.3–3.4) for ECG-gated and 3.3 mSv (2.0–4.2) for non–ECG-gated cardiac CT.

Cardiac CT detected 254 thrombi in 243 (6.2%) patients. Cardiac thrombi were located in the LAA (n=193, 4.9%), LA (n=19, 0.5%), and LV (n=42, 1.1%; Table 2; Table S8). A cardiac thrombus was detected on ECG-gated cardiac CT in 111 of 1457 (7.6%) patients and on non–ECG-gated cardiac CT in 132 of 2462 (5.4%). Of the total study population, 1342 (34%) patients underwent echocardiography. The median interval between cardiac CT and echocardiography was 9 (IQR, 1–57) days. Among 1323 patients who underwent both cardiac CT and TTE, 93 had a cardiac thrombus detected on cardiac CT, 19 (20%) of which were subsequently identified on TTE (Table 3). The diagnostic yield of cardiac CT was higher than that of TTE: OR, 7.4 (95% CI, 4.0–15.1), *P*<0.001. TTE detected 10 thrombi that were not detected on cardiac CT, located in the LA (n=1) and LV (n=9). In 4 patients with an LV thrombus that was not detected on cardiac CT, the CT scan window did not include the LV. Among 126 patients who underwent cardiac CT and TEE, 5 cardiac thrombi were detected on cardiac CT, of which 2 (40%) were identified on TEE (Table S9).

**Table 2.**
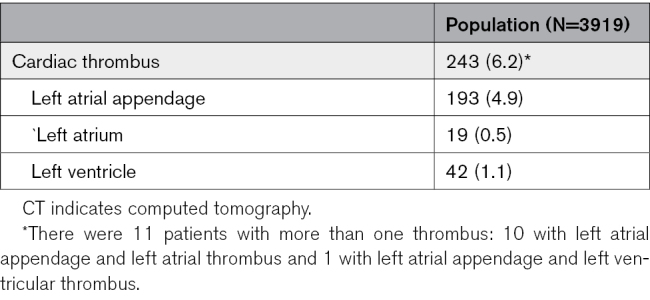
Cardiac Thrombi Detected on Cardiac CT

**Table 3.**
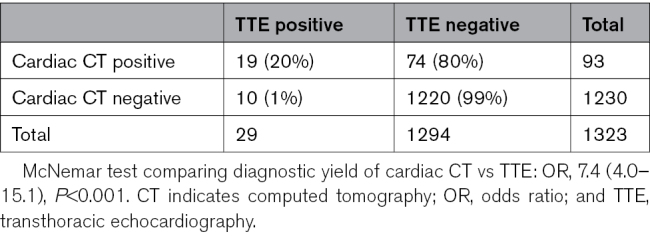
Cardiac Thrombi Detected on Cardiac CT Versus TTE

Patients with a cardiac thrombus had a worse functional outcome at 90 days than patients without a thrombus (median mRS score, 3 [IQR, 2–6] versus 2 [IQR, 1–4], adjusted OR [aOR], 1.6 [95% CI, 1.2–2.0]; Figure S3; Table 4) and a higher mortality: 33% versus 15%, aOR, 1.6 (95% CI, 1.1–2.3). There was a higher risk of MACE in patients with a cardiac thrombus (28% versus 13%, aOR, 1.4 [95% CI, 1.0–2.0]; Table S10). Recurrent stroke rates did not differ between the groups: 5% versus 4%, aOR, 1.4 (95% CI, 0.7–2.5). Including hospital location as a random effect showed similar results (Table S11). Patients with a cardiac thrombus had higher rates of AF de novo during hospitalization compared with patients without a cardiac thrombus (20% versus 9%, *P*<0.001) and received a final diagnosis of AF more often (78% versus 32%, *P*<0.001; Table S12). AF was never detected in 54 (22%) of patients with a cardiac thrombus.

**Table 4.**
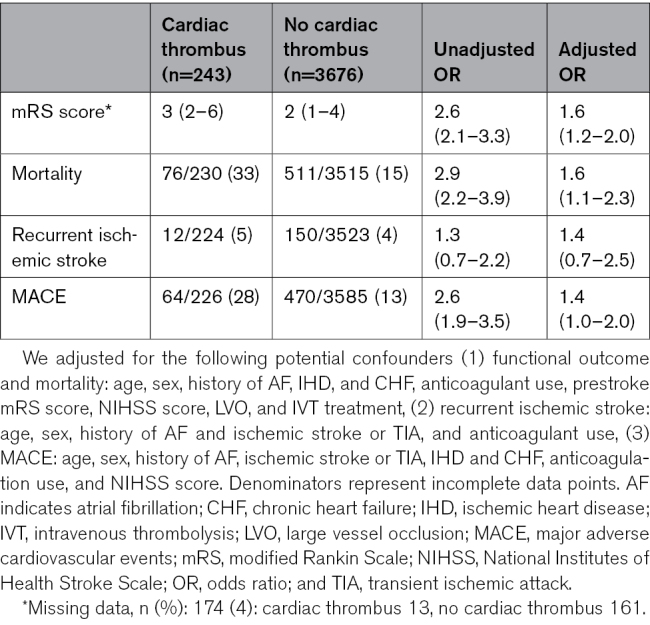
Outcomes at 90-Day Follow-Up

Cardiac CT appeared to influence patient management in terms of initiation of anticoagulation in those with a detected cardiac thrombus. For instance, anticoagulation was started in 31 of 54 (57%) patients without a history of AF and without AF detected during follow-up (Table S13). Moreover, in 35 of 61 (57%) patients with a history of AF and no anticoagulation at baseline, and 3 of 4 patients in whom AF was detected during follow-up, anticoagulation was initiated. The most important reason for not initiating anticoagulation in patients with a cardiac thrombus was in-hospital death. Overall, 132 of 155 (85%) patients with a cardiac thrombus detected on CT and who were alive at discharge received anticoagulation.

Subgroup analyses showed that cardiac thrombi were more often detected in older patients (≤74 years: 4% versus >74: 8%, *P*<0.001), women (female patients: 7% versus male patients: 5%, *P*=0.019), patients with a history of AF (yes: 15% versus no: 4%, *P*<0.001), LVO (yes: 9% versus no: 5%, *P*<0.001), and a higher NIHSS score (NIHSS score <5: 3% versus NIHSS score 5–10: 5% versus NIHSS score >10: 11%, *P*<0.001; Figure).

**Figure.**
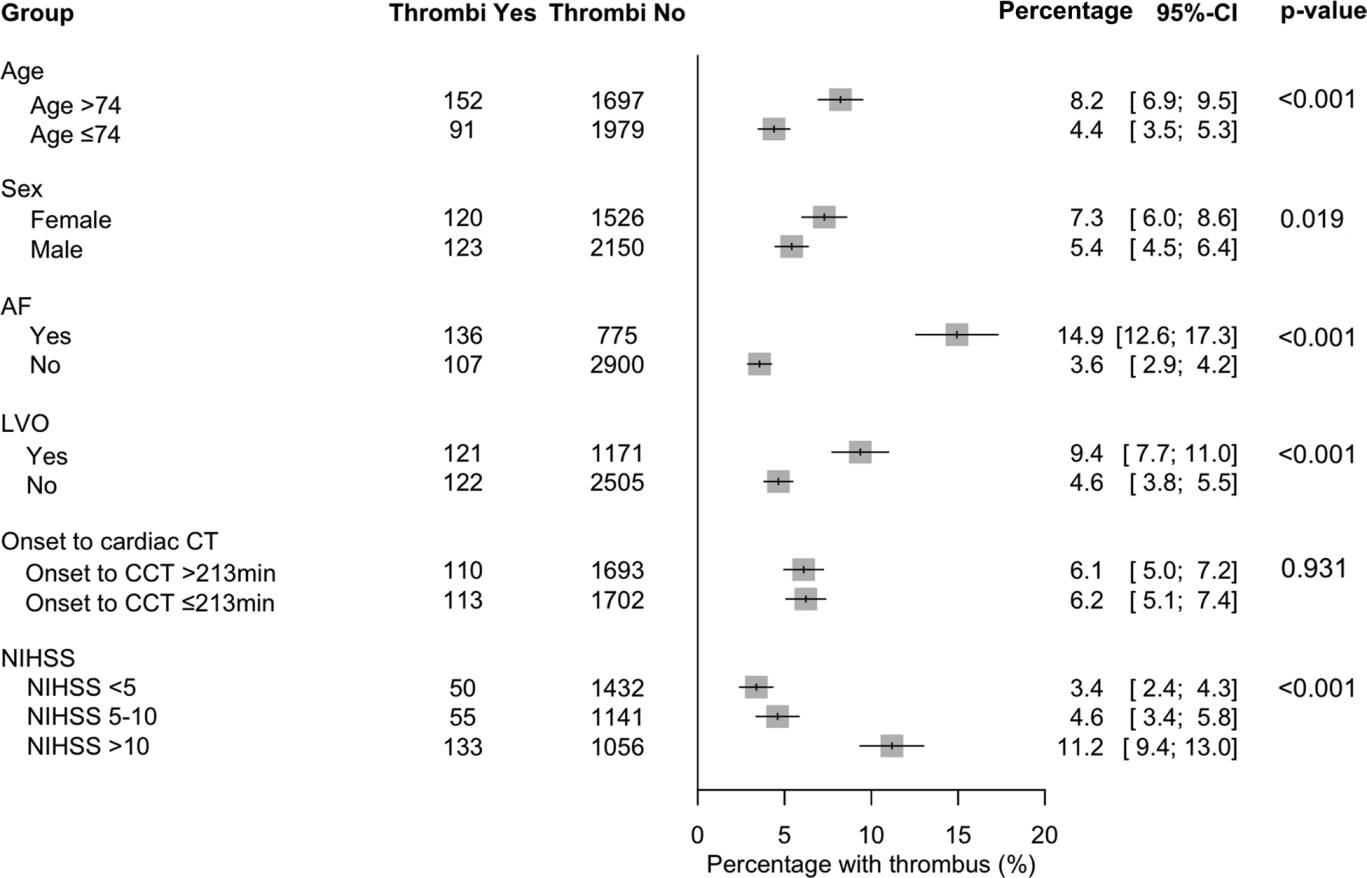
**Forest plot of subgroup analysis and risk of cardiac thrombus detection.** AF indicates atrial fibrillation; CT, computed tomography; CCT, cardiac computed tomography; LVO, large vessel occlusion; and NIHSS, National Institutes of Health Stroke Scale.

## Discussion

This multicenter study among almost 4000 patients with AIS shows that cardiac CT acquired as part of the acute scan protocol is feasible and that it detects a cardiac thrombus in ≈6% of patients. The diagnostic yield of cardiac CT to detect cardiac thrombi was substantially higher compared with TTE performed as part of the standard stroke workup. The presence of a cardiac thrombus was associated with a worse functional outcome, a higher risk of MACE, and a higher mortality at 90-day follow-up, but not with a higher risk of recurrent stroke.

In our study, cardiac CT clearly outperformed TTE, most likely due to its higher diagnostic accuracy, as visualizing the LAA on TTE is challenging.^6^ However, it is important to consider that the interval between the 2 modalities was 9 days, which increases the possibility that cardiac thrombi may have dissolved before the TTE was performed.^13^ In the Mind the Heart study, however, the median interval between cardiac CT and TTE was only one day, and the difference in yield between CT and TTE was similar. The higher diagnostic yield of cardiac CT compared with echocardiography was also confirmed in 2 meta-analyses including 43 studies^8^ and 14 studies.^14^ These findings indicate that cardiac CT improves the ability to detect cardiac thrombi in patients with AIS. However, echocardiography may still play an important role for indications, such as the detection of patent foramen ovale,^15^ assessment of cardiac function,^6^ and detection of valvular abnormalities.

Ten cardiac thrombi detected on TTE were not detected on cardiac CT, of which 4 LV thrombi were not visualized on cardiac CT because the scan window did not include the LV. The remaining 6 thrombi could be false negatives on cardiac CT; however, 2 meta-analyses reported a low risk of false negatives on CT with rates of 1% and 0.2%, respectively.^8,14^ In addition, there may be a risk of false positives on TTE, particularly when contrast is not used, due to prominent trabeculations or anatomic variations of the LV, resulting in a low positive predictive value.^16–18^ Overall, these cases were uncommon, and the diagnostic yield of cardiac CT remains higher than TTE.

The presence of a cardiac thrombus was associated with a worse clinical outcome. Patients with cardioembolic stroke generally have worse outcomes, mostly driven by the fact that they suffer more severe strokes.^4^ However, we took this into account by adjusting the analyses for NIHSS score and LVO. The higher mortality could be related to a higher cardiovascular burden, as supported by the higher rate of MACE observed in our study in patients with a cardiac thrombus. Another hypothesis is that patients with a cardiac thrombus have an increased risk of recurrent stroke while not being treated with anticoagulation in the acute phase, although this hypothesis is not supported by the data in our study. It should be noted, however, that the observational design, absence of routine follow-up imaging, and reliance on routine clinical practice may have led to an underestimation of recurrent stroke rates. Furthermore, patients with a cardiac thrombus were likely treated with anticoagulation either due to known or newly detected AF, or because thrombus detection itself possibly prompted the initiation of anticoagulants, which may have reduced the risk of recurrent stroke.

Currently, there is no randomized clinical evidence to support the optimal treatment once a cardiac thrombus is detected in patients with AIS and without known AF. For patients with an LV thrombus, current guidelines recommend anticoagulation, but for LAA and LA thrombi without AF, cardiomyopathy and LV dysfunction, the optimal therapy is not specified.^19,20^ When a thrombus is detected in the absence of AF and AF is not identified during hospital admission, it is generally common for clinicians to initiate anticoagulation. Early detection of a cardiac thrombus provides an opportunity to commence anticoagulation at an early stage, underscoring the clinical relevance of early thrombus detection, especially because recent studies have shown that early initiation of anticoagulation is safe in most patients with AIS.^21^ Cardiac CT may also reduce treatment delays in initiating anticoagulation in patients in whom AF is detected later during hospital admission or follow-up, which could prevent recurrent ischemic stroke.

Approximately half of the patients with a cardiac thrombus had a history of AF and therefore already had an indication for anticoagulation therapy. However, detecting a cardiac thrombus may still provide clinically relevant information for these patients. Patients with a cardiac thrombus had worse functional outcomes and higher mortality at follow-up despite prior anticoagulation use, suggesting that there is potentially room for improved management in these patients. Furthermore, the occurrence of ischemic stroke in patients with known AF may indicate nonadherence or inadequate therapy, and the detection of a thrombus could prompt clinicians to modify or intensify the current therapy. This is supported by an international survey among 402 neurologists, in which 93% of respondents indicated that detecting a LAA thrombus would influence secondary prevention strategies even in patients with AF.^5^

Two of the 4 hospitals in this study used ECG-gated scans, whereas the other 2 used a nongated technique. Implementation of non–ECG-gated scans is generally easier, as ECG-gated cardiac CT requires specific hardware and software and often requires repositioning of the patient before the heart is imaged. This is reflected by the longer scan time for ECG-gated than non-gated scans (6 minutes versus 13 seconds). In contrast, ECG-gated scanning reduces the risk of motion artifacts by synchronizing image acquisition with the cardiac rhythm.^22,23^ Future studies should focus on directly comparing different cardiac CT acquisition protocols in patients with AIS.

The additional radiation dose of cardiac CT and DLP in this study were comparable to the literature,^24–26^ which indicates a range of 0.91 to 5.10 mSv in non–ECG-gated^9,10,26–28^ and 1.52 to 1.67 mSv in ECG-gated cardiac CT.^29,30^ When compared with other components of the stroke imaging protocol, cardiac CT had a median DLP between 141 and 239, whereas the DLP for the standard CTA of the intracranial arteries and carotids typically ranges from 584 to 1416 and for CT perfusion from 995 to 1916, depending on the scan protocol.^31,32^

Our reported rate of cardiac thrombi is slightly lower than 2 previous studies. The recent DAYLIGHT study (The Extended CTA for the Successful Screening of Cardioaortic Thrombus in Acute Ischemic Stroke and TIA) compared an extended (at least 6 cm below carina) non–ECG-gated CTA scan protocol versus standard CTA in a randomized controlled study design in 465 patients with AIS and TIA.^9^ They detected a total of 18/226 (8%) cardiac thrombi in the LAA, LA, and LV. The ENCLOSE study (Improved Prediction of Recurrent Stroke and Detection of Small Volume Stroke) investigated non–ECG-gated cardiac CT in patients with AIS and TIA and reported a cardiac thrombus in 44 of 370 (12%) patients. The slightly lower detection rate in our study may partly be explained by patient selection or because both DAYLIGHT and ENCLOSE used a non–ECG-gated protocol without a delayed scan. A delayed scan provides additional time-resolved information about contrast to reach the LAA. Omitting a delayed scan can make it more difficult to differentiate between a thrombus and atrial slow-flow, which is commonly encountered in patients with AF.^6,33,34^ Both studies also showed a higher detection rate of cardiac thrombi compared with TTE, similar to our study.

A cardiac thrombus was more often detected in older patients, women, those with a history of AF, presence of LVO, and a higher baseline NIHSS score. Future studies should provide an in-depth analysis of the diagnostic yield of cardiac CT in specific subgroups. Such insights could help to identify which patients benefit most from cardiac CT and in which patients it may be considered to skip the cardiac CT because of the low yield. In addition, developing a prediction model using baseline characteristics could help guide clinicians in selecting patients for cardiac CT based on the expected yield for detecting a thrombus.

Our study has several limitations. First, there was heterogeneity between the participating hospitals in scan protocol and cardiac thrombi definition. In 31%, the LV was not visualized on cardiac CT, likely resulting in some missed thrombi. John Hunter Hospital included patients with higher NIHSS scores during the last 16 of 44 months (36%) of their inclusion period, which may have introduced selection bias. However, as this study reflects routine clinical practice, it does enhance the external variability of the findings. Second, we did not include patients with TIA, and because these patients generally have milder symptoms, the yield of cardiac CT may not directly apply to this group. Third, echocardiography was only performed in approximately one-third of patients. Fourth, our methodology of follow-up largely relied on reviewing medical files. Although we complemented data from the medical records with information gathered by contacting patients, we still may have underestimated events. Fifth, we did not have a core lab committee to assess all cardiac CT scans. To improve accuracy, all the cardiac thrombi and filling defects suspected of a thrombus were assessed by an experienced cardiac radiologist or cardiac imaging cardiologist. Last, due to the observational study design, residual confounding cannot be fully excluded in the outcome analyses, despite adjusting for factors such as NIHSS score and LVO.

In summary, this study demonstrates that implementing cardiac CT into the routine acute stroke imaging protocol is feasible, that it results in detection of a cardiac thrombus in ≈6% of patients and that it has a 7× higher diagnostic yield than TTE. Patients in whom a cardiac thrombus was identified had a higher risk of death and a worse functional outcome.

## ARTICLE INFORMATION

### Sources of Funding

### Disclosures

The author(s) declared the following potential conflicts of interest with respect to the research, authorship, and publication of this article: Dr Rinkel has received a grant from the Hartstichting. Dr Green received a grant from the Australian Government Research Training Program Scholarship. Dr Garcia-Esperon has received travel funding for travel for conferences from Boehringer Ingelheim and Bayer, speaker honoraria and compensation for consultant services from AstraZeneca, and compensation for consultant services from Basking Biosciences, outside the submitted work. Dr Berry-Noronha has received research funding from the Neurological Foundation of New Zealand, outside the submitted work. Prof Coutinho reports grants from AstraZeneca and Bayer, and compensation for consultant services from Portola Pharmaceuticals LLC, outside the submitted work (all paid to institution). Prof Coutinho is co founder and shareholder of Founder and shareholder of TrianecT BV. The other authors report no conflicts.

### Supplemental Material

Tables S1–S13

Figures S1–S3

MOOSE Guidelines

## Supplementary Material
