# Supplementary material for: Diagnostic Yield of Cardiac CT to Detect Cardiac Thrombi in Patients With Acute Ischemic Stroke (AIS of HEARTS)
Source: Stroke. 2026 Jun 2;57(8):2408–16. doi: 10.1161/STROKEAHA.126.055575 (PMC13399728; doi:10.1161/STROKEAHA.126.055575)
Supplement: Supplementary file 1 [file str-57-2408-s001.pdf]

Supplemental material: Diagnostic Yield of Cardiac CT to Detect Cardiac Thrombi in Patients with Acute Ischemic Stroke (AIS of HEARTS).

Table S1. AIS of HEARTS collaboration: aims and steering committee

Table S2. List of variable definitions AIS of HEARTS

Table S3. Acute stroke scan protocol per center

Table S4. Details of participating hospitals

Table S5. Definition of cardiac thrombus per hospital

Table S6. Baseline characteristics per center

Table S7. Cardiac CT scan parameters

Table S8. Detected cardiac thrombi per center

Table S9. Cardiac thrombus detected on cardiac CT versus TEE

Table S10. Major Adverse Cardiovascular Events (MACE) per item

Table S11. Outcomes at 90-day follow-up including hospital location as random effect

Table S12. Atrial fibrillation detected per phase

Table S13. Anticoagulation use in patients with a cardiac thrombus detected on cardiac CT

Figure S1. Schematic representation of non-ECG-gated and ECG-gated scan protocols per center

Figure S2. Flowchart

Figure S3. Modified Rankin Scale (mRS)-scores at 90 days

Table S1. AIS of HEARTS collaboration: aims and steering committee

The aim of the AIS of HEARTS registry is:<br>*To establish a database to collect, maintain and provide accessible individual patient data from international cohorts and to provide pooled analyses about the yield and value of cardiac computed tomography (CT), acquired during the initial stroke imaging protocol.*	
**Steering committee**	
Amsterdam UMC	Prof. Dr. J. Coutinho
Christchurch Hospital	Dr. T.Y. Wu
John Hunter Hospital	Dr. C. Garcia-Esperon
	Prof. Dr. N.J. Spratt
Liverpool Hospital	Prof. Dr. M.W. Parsons

Table S2. List of variable definitions AIS of HEARTS

Variable	Definition
Acute ischemic stroke	Acute onset neurological deficits with either corresponding lesions on brain imaging (CT or MRI) indicative of AIS or a high clinical suspicion. In cases of uncertainty, a neurologist reviewed these patients.<br>A selection of patients underwent brain MRI to verify the diagnosis of ischemic stroke.
Large vessel occlusion	Occlusion of: intracranial internal carotid artery, the proximal segment of the middle cerebral artery (M1 and proximal M2), or basilar artery.
Ischemic heart disease	Medical history of myocardial infarction, past coronary artery bypass graft surgery or angina pectoris.
Recurrent ischemic stroke	New acute onset neurological deficits, with either accompanying lesions on imaging or a high clinical suspicion. Re-occlusions after EVT were considered as recurrent strokes. If new stroke was identified on imaging (CT or MRI) and was asymptomatic it was not considered recurrent stroke.
Transient ischemic attack	New acute onset neurological deficits that recover <24 hours, without lesions on imaging indicative of ischemic stroke.
Intracerebral hemorrhage	Hemorrhage based on the Heidelberg bleeding classification.[29] Excluding traumatic intracranial hemorrhages.
Major bleeding	Fatal bleeding, and/or symptomatic bleeding in a critical area or organ, such as intracranial, intraspinal, intraocular, retroperitoneal, intra-articular or pericardial, or intramuscular with compartment syndrome, and/or bleeding causing a fall in hemoglobin levels of 1.24 mmol/L (20 g/L or greater) or more, or leading to a transfusion of 2 U or more of whole blood or red cells.[30] Including traumatic hemorrhages, excluding intracerebral hemorrhage (defined above).
Cardiac events	Atrial fibrillation, myocardial infarction, cardiac failure.
Systemic arterial occlusion	Occlusion located elsewhere than intracranial.
Venous thromboembolism	Thrombosis in any venous location. Including deep vein thrombosis and pulmonary embolism.
Unspecified stroke	Patients with neurological complaints suspect of a stroke who did not consult medical advice.
MACE	Composite of non-fatal ischemic stroke, non-fatal myocardial infarction, and cardiovascular death.
Stroke etiology: incomplete evaluation	Patients that did not receive one of the following: cardiac CT, ECG or 24-hour rhythm monitoring.
Stroke etiology: Other - PFO	PFO was considered the etiology of stroke based on the ROPE criteria.

AIS=Acute Ischemic Stroke, CT=Computed Tomography, MRI=Magnetic Resonance Imaging, ECG=Electrocardiogram, PFO=Patent Foramen Ovale, ROPE=Risk of Paradoxical Embolism.

Table S3. Acute stroke scan protocol per center

**John Hunter Hospital**<br><br>All patients were examined using a 384 (2 x 192) slice Dual Source CT scanner (SOMATOM Force, Siemens Healthcare, Forchheim, Germany or GE VCT Lightspeed, GE Healthcare, Waukesha, WI, USA). SOMATOM scan parameters for the CTP were using 192×0.6mm collimation, 0.25 s rotation time, 70 kV (kilo voltage) tube voltage and 450mAs (milliampere-seconds) effective tube current time product. Contrast enhanced CT perfusion scans were acquired using 50 ml Omnipaque 350 (GE Healthcare, WI, USA), 50ml bolus sodium chloride and an injection rate of 6 ml/s. A soft spatial resolution kernel (Hr36) was chosen to reconstruct the data with 5mm slice thickness in a 512×512 image matrix and data was automatically transferred to MiStar for analysis. The CTA Circle of Willis – Carotids acquisition was obtained in the caudocranial direction, with 128×0.6mm collimation, 0.25 s rotation time, 1.0 Pitch, 120 kV tube voltage and 350mAs effective tube current time product. Contrast enhanced CTA images were acquired using 50 ml Omnipaque 350 (GE Healthcare, WI, USA), 50ml bolus sodium chloride and an injection rate of 6 ml/s. A soft spatial resolution kernel (Bv36) was chosen to reconstruct the data with 1.0mm slice thickness in a 512×512 image matrix and data was automatically transferred to PACS and Syngo Via. CTA scan range included from aortic arch inferiorly to skull vertex superiorly. Acquisition parameters for the cardiac CT were 120/120 kV tube voltage and 206mAs. The cardiac CT was scanned at 30 seconds and again 2 minutes from the start of the 2nd contrast injection.
**Christchurch Hospital**<br><br>All patients were examined using either a 128 or 64-slice MDCT scanner (SOMATOM Definition Flash, Siemens Healthcare, Forchheim, Germany or GE VCT Lightspeed, GE Healthcare, Waukesha, WI, USA). SOMATOM scan parameters for the CTP were using 128×0.6mm collimation, 0.285 s rotation time, 70 kV tube voltage and 250mAs effective tube current time product. Contrast enhanced CT perfusion scans were acquired using 40 ml Omnipaque 300 (GE Healthcare, WI, USA), 30ml bolus sodium chloride and an injection rate of 6 ml/s. A soft spatial resolution kernel (H20f) was chosen to reconstruct the data with 5mm slice thickness in a 512×512 image matrix and data was automatically transferred to MiStar for analysis. The CTA Circle of Willis – Carotids acquisition was obtained in the craniocaudal direction with 128×0.6mm collimation, 0.285 s rotation time, 1.2 Pitch, 120 kV tube voltage and 300mAs effective tube current time product. Contrast enhanced CTA images were acquired using 60 ml Omnipaque 300 (GE Healthcare, WI, USA), 30ml bolus sodium chloride and an injection rate of 5 ml/s. A soft spatial resolution kernel (i30f) was chosen to reconstruct the data with 0.6mm slice thickness in a 512×512 image matrix and data was automatically transferred to PACS. CTA scan range included superior to the anterior cerebral arteries and inferior to the left atrium. A second auto planned cardiac CT was performed 10 seconds after completion of the CTA acquisition to cover the LAA. Acquisition parameters for the cardiac CT were 120 kV tube voltage and 110mAs effective tube current time product.
**Amsterdam UMC**<br><br>All patients were examined using, a third-generation Dual Source CT scanner (Somatom Force, Siemens Healthineers, Erlangen, Germany). After history talking and physical examination, patients sequentially underwent non–contrast-enhanced CT of the brain, CT perfusion, and non-gated CT-angiography of the aortic arch, cervical, and intracranial arteries. A non-contrast CT brain was initially acquired at a fixed 120 kVp (kilo voltage peak). Contrast enhanced CT perfusion scans were acquired using 35 mL (85% contrast and 15% Saline) of contrast bolus (Xenetix 350mgl/ml), 40ml bolus chaser of sodium chloride, both at an injection rate of 6 ml/s. CTP scan parameters were 48×1.2mm collimation, 0.25 s rotation time, 70 kV tube voltage and 200mAs effective tube current time product. The scan range or z-axis for the perfusion was 114 mm. A CTA Carotids-head

acquisition was acquired using 50 mL bolus (85% contrast 15% NaCl) of Xenetix 350mgl/ml, 40ml bolus chaser of sodium chloride at an injection rate of 6 ml/s. The CTA Circle of Willis – Carotids was obtained in a caudocranial direction with 128×0.6mm collimation, 0.25 s rotation time, 1.2 Pitch, 80-100 kV tube voltage and 200mAs effective tube current time product. CTA scan range included from aortic arch inferiorly to skull vertex superiorly. Approximately five minutes after completion of the CT angiogram, a third dose of iodinated contrast (40 mL of Xenetix 350mgl/ml) was administered followed by a 40ml bolus chaser of sodium chloride both at an injection rate of 6 ml/s. Contrast was injected in the right arm, preferred cubital vein with an 18-gauge intravenous catheter. A prospectively gated cardiac CT scan in a cranial-caudal direction was then triggered using the timing of the a. cerebri media which was calculated from the CT perfusion minus 2 seconds of scan delay as timing for start gated cardiac CT. Cardiac CT scan parameters were 152×0.6mm collimation, 0.25 s rotation time, 100 kV tube voltage and 288mAs effective tube current time product. Imaging was obtained during 63-76% diastole. The patient's arms were positioned adjacent to their head during the cardiac CT scan. Axial cardiac CT images were reconstructed with a section thickness of 0.6 mm and increment of 0.4 mm, with a standard kernel with true fidelity Bv40. Pharmacological agents, including nitrates and beta blockers, were not administered. If alteplase was ready for administration before hyperacute cardiac CT, IVT could be initiated before acquiring cardiac CT. Radiation dose in the dose length product was recorded.
**Liverpool Hospital**<br><br>All patients were examined using a 256-slice CT scanner (GE RevolutionTM Apex Platform). A non-contrast CT brain was initially undertaken using a fixed 120 kVp technique. A first dose of iodinated contrast (50 mL of Omnipaque 350) was then administered to facilitate a CT cerebral perfusion study, followed by a 50 mL normal saline flush. The scan range or z-axis for the perfusion was 120 mm and acquisition parameters were 80 kVp and 115 mAs. Two to three minutes after this first contrast dose, a second dose of iodinated contrast (50 mL of Omnipaque 350) was administered followed by a 50 mL normal saline flush. A CTA of the aortic arch to the Circle of Willis was then obtained using bolus tracking and with a tube voltage between 80 – 120 kVp. Approximately three minutes after completion of the CT angiogram, a third dose of iodinated contrast (70 mL of Omnipaque 350) was administered using a split bolus technique followed by a 50 mL normal saline flush. A prospectively gated cardiac CT scan was then triggered using a bolus tracking technique with a trigger set at the ascending aorta. Imaging was obtained during diastole. If the heart rate exceeded 71 bpm, imaging during systole was also obtained to improve yield due to motion artefact. The patient's arms were positioned during the cardiac CT scan adjacent to their head. Secondary reconstruction of systolic imaging was undertaken using SnapShot Freeze, a GE software, in conjunction with the GE Advanced Workstation (AW) server. The tube voltage was 80 to 120 kV. Axial images for the cardiac CT were reconstructed with a section thickness of 0.625 mm and an increment of 0.625 mm, with a standard kernel with true fidelity. Pharmacological agents, including nitrates and beta blockers, were not administered in view of the time-critical nature of imaging acquisition and to avoid hypotension in the setting of acute stroke.

Table S4. Details of participating hospitals.

	Christchurch Hospital	Amsterdam UMC	John Hunter Hospital	Liverpool Hospital
**Sample size**	1739	1394	723	63
**City-Country**	Christchurch-New Zealand	Amsterdam, The Netherlands	Newcastle, Australia	Sydney, Australia
**Patient population**	Patients with AIS. Symptoms <24h.	Patients with AIS. Symptoms <24h.	Patients with AIS. Symptoms <24h.	Patients with AIS. Symptoms <24h.
**Inclusion period**	September 2018 – June 2024	May 2018 – June 2024	November 2020 – June 2024	October 2022 – June 2024
**Cardiac CT protocol**	Non-ECG-gated cardiac CT. Since January 2020 delayed scan, 10 seconds after CTA.	ECG-gated, prospective. Mimicking delayed scan by using contrast from CTP/CTA.	Non-ECG-gated cardiac CT. Delayed scan 30 seconds and 2 minutes after CTA.	ECG-gated, prospective. Mimicking delayed scan by using contrast from CTP/CTA.
**Indication for cardiac CT**	Patients with acute neurological symptoms, duration <24h.<br>Delayed scan not performed in patients age ≤50.	Patients with acute neurological symptoms, duration <24h.	Patients with acute neurological symptoms, duration <24h.<br>Since March 2023 cardiac CT only performed in patients with NIHSS≥4.	Patients with acute neurological symptoms, duration <24h. Dependent on availability and expertise of radiographers.
**Cardiac CT assessment (primary outcome)**	First assessment by on call radiologist. All scans assessed by PhD student. All filling defects assessed by cardiac radiologist.	First assessment by on call radiologist. Partly completely assessed by cardiac radiologist (n=444); remaining cases assessed by PhD student and filling defects assessed by cardiac radiologist.	First assessment by on call radiologist. Second assessment by neurologist. All filling defects assessed by cardiac radiologist.	First assessment by imaging cardiologist. All scans were assessed by a PhD student, cardiac radiologist, and a cardiac imaging specialist cardiologist.
**Echocardiography**	Assessment by local cardiologist.	Assessment by local cardiologist.	Assessment by local cardiologist.	Assessment by local cardiologist.
**Follow-up**	No routine follow-up. 90-day and 2-year follow-up data retrieved from medical files. Patients from Christchurch hospital would likely return there for follow-up events.	Standard telephone visit neurology after 90 days. Additional data and 2-year follow-up with telephone calls to patient/general practitioner/other hospitals or retrieved from medical files.	Standard outpatient clinic visit neurology after 90 days. Additional data and 2-year follow-up with telephone calls to patients or retrieved from medical files.	Follow-up as directed by treating physician. 90-day mRS data retrieved from medical files.<br>No 2-year follow-up data.

			John Hunter Hospital had access to the medical records of most hospitals across New South Wales, Australia.	

AIS=Acute Ischemic Stroke, ECG=Electrocardiogram, CTP = Computed Tomography Perfusion, CTA = Computed Tomography Angiography, NIHSS=National Institutes of Health Stroke Scale, LVO = Large Vessel Occlusion, ED = Emergency Department, mRS=Modified Rankin Scale, TTE=Transthoracic Echocardiography, TEE=Transesophageal Echocardiography.

Hospital	Definition
**Christchurch Hospital**	A filling defect <100 Hounsfield Unit (HU) present on first and delayed cardiac CT with a well-defined ovoid/round shape not caused by motion artifact or atrial trabeculae.
**John Hunter Hospital**	A filling defect present on first and delayed cardiac CT.
**Amsterdam UMC**	A filling defect that appears as a low-attenuated mass (typically <100 HU) and an oval or round shape.
**Liverpool Hospital**	A filling defect that appears as a low-attenuated mass (typically <100 HU) and an oval or round shape.

Table S5. Definition of cardiac thrombus per hospital

Table S6. Baseline characteristics per center

	Christchurch Hospital (N=1739)	Amsterdam UMC (N=1394)	John Hunter Hospital (N=723)	Liverpool Hospital (N=63)
**Age – year (IQR)**	75 (65-83)	72 (62-81)	74 (62-81)	75 (64-82)
**Sex – male, n (%)**	990 (57)	795 (57)	457 (63)	31 (49)
**Medical history, n (%)**				
**Previous stroke or TIA**	486 (28)	363/1393 (26)	127 (18)	16 (25)
**Atrial fibrillation**	498 (29)	233/1393 (17)	160 (22)	20 (32)
**Diabetes Mellitus**	362 (21)	295 (21)	170 (24)	20 (32)
**Hypertension**	1095 (63)	684 (49)	421 (58)	45 (71)
**Hypercholesterolemia**	610 (35)	203/1393 (15)	232 (32)	36 (57)
**Smoking**				
**Never smoked**	1072/1731 (62)	654/1275 (51)	279/599 (47)	47/61 (77)
**Current smoker/past 6 months**	177/1731 (10)	314/1275 (25)	153/599 (26)	6/61 (10)
**Previous smoker/>6 months**	482/1731 (28)	307/1275 (24)	167/599 (28)	8/61 (13)
**Malignancy - active**	80 (4)	69 (5)	37 (5)	3 (5)
**Ischemic Heart Disease**	448 (26)	249/1392 (18)	118 (16)	17 (27)
**Chronic heart failure**	250 (14)	76/1393 (5)	59 (8)	6 (10)
**Pre-stroke mRS-score (IQR)***	0 (0-1)	0 (0-1)	0 (0-1)	1 (0-2)
**Medication, n (%)**				
**Anticoagulation**	269 (15)	242 (17)	129 (18)	13 (21)
**DOAC**	204 (12)	114 (8)	93 (13)	13 (21)
**Vitamin K antagonist**	57 (3)	48 (3)	30 (4)	0
**INR<2.0**	29/57 (51)	22/48 (46)	22/30 (73)	0
**Antiplatelet**	657 (38)	426 (31)	145 (20)	26/62 (42)
**Systolic blood pressure, mmHg (IQR)[†]**	158 (139-179)	152 (135-172)	150 (132-169)	139 (122-156)
**Diastolic blood pressure, mmHg (IQR)[‡]**	83 (76-95)	85 (74-97)	80 (72-90)	82 (74-93)
**NIHSS (IQR)[§]**	6 (4-12)	5 (2-12)	7 (3-15)	9 (4-17)

**Large Vessel Occlusion, n (%)**	601 (35)	415 (30)	249 (34)	27 (43)
**Reperfusion therapy, n (%)**				
**Intravenous thrombolysis**	540/1731 (31)	525 (38)	127 (18)	18 (29)
**Endovascular thrombectomy**	448 (26)	276 (20)	197 (27)	20 (32)
**Diagnostic procedures, n (%)**				
**ECG**	1722 (99)	1350 (97)	723 (100)	63 (100)
**Rhythm monitoring >24 hours**	384 (22)[#]	1258/1353 (93)	614 (85)	63 (100)
**Echocardiography**	294 (17)	703 (50)	300 (42)	45 (71)
**TTE**	287 (17)	700 (50)	293 (41)	43 (68)
**TEE**	50 (3)	31 (2)	37 (5)	8 (13)
**Time cardiac CT until echocardiography (days)**[||]	12 (3-87)	9 (1-47)	10 (3-85)	3 (2-5)
**Brain MRI**	483 (28)	179 (13)	435 (60)	47 (75)

IQR=Interquartile Range, TIA=Transient Ischemic Attack, mRS=Modified Rankin Scale, DOAC=Direct Oral Anticoagulant, INR=International Normalized Ratio, NIHSS=National Institutes of Health Stroke Scale, ECG=Electrocardiogram, TTE=Transthoracic Echocardiography, TEE=Transesophageal Echocardiography, MRI=Magnetic Resonance Imaging. Denominators represent incomplete data points.

Large Vessel Occlusion includes: internal carotid artery, M1, proximal M2 and basilar artery.

Missing data, n (%): *31 (0.8): Christchurch 6, Amsterdam UMC 25, †226 (6): Christchurch 220, Amsterdam UMC 3, John Hunter 1, Liverpool 2, ‡283 (7): Christchurch 277, Amsterdam UMC 3, John Hunter 1, Liverpool 2, §52 (1): Christchurch 50, John Hunter 2, ||7 (0.2): Amsterdam UMC 6, John Hunter 1.

[#] Although patients at Christchurch Hospital underwent rhythm monitoring, documentation of whether these had been reviewed was inconsistent.

Table S7. Cardiac CT scan parameters

	ECG-gated (N=1457)	Non-ECG-gated (N=2462)
**Heart completely imaged, n (%)**	1447 (99)	1273 (52)
**Delayed scan performed, n (%)**	NA	2006 (81)
**Duration cardiac CT, seconds**	4	NA‡
**Additional scan time (end CTA, end cardiac CT)***	6 (5-7) min	13 (12-61) sec
**Dose-length product cardiac CT, mGy*cm (IQR)†,§**	141 (93-242)	239 (144-303)
**Additional radiation exposure, mSv (IQR)**	2.0 (1.3-3.4)	3.3 (2.0-4.2)

CT=Computed Tomography, ECG=Electrocardiogram, LAA=Left Atrial Appendage, mGy*cm=Milligray-centimeter, mSv=Millisievert.
Missing data, n (%): *92 (2): ECG-gated 88, Non-ECG-gated 4, †36 (0.9): ECG-gated 16, Non-ECG-gated 20.
‡We do not have data on scan duration of non-ECG-gated cardiac CT, but it was estimated at 1 second.
§At Christchurch Hospital, the DLP value for the first cardiac CT was unavailable because it was an extension of the CTA. Therefore, we estimated the DLP by dividing the CTA DLP by the length of the cardiac CT segment (from below aortic arch, until end of CTA).

**Hospital**	**Christchurch Hospital (N=1739)**	**Amsterdam UMC (N=1394)**	**John Hunter Hospital (N=723)**	**Liverpool Hospital (N=63)**
**Cardiac thrombus (N=243)**	104 (6%)	95 (7%)	28 (4%)	16 (25%)

Table S8. Detected cardiac thrombi per center

Table S9. Cardiac thrombi detected on cardiac CT versus TEE

	TEE positive	TEE negative	Total
**Cardiac CT positive**	2 (40%)	3 (60%)	5
**Cardiac CT negative**	0 (0%)	121 (100%)	121
**Total**	2	124	126

CT=Computed Tomography, TEE=Transesophageal Echocardiography.
McNemar test to compare diagnostic yield between cardiac CT and TEE not possible because one group has the value of zero.

Table S10. Major Adverse Cardiovascular Events (MACE) per item

	Cardiac thrombus (n=243)*	No cardiac thrombus (n=3676)†
Fatal hemorrhagic stroke	2/226 (0.9)	42/3585 (1.2)
Fatal ischemic stroke	52/226 (23.0)	248/3585 (6.9)
Fatal cardiac cause	1/226 (0.4)	36/3585 (1.0)
Non-fatal myocardial infarction	4/226 (1.8)	31/3585 (0.9)
Non-fatal ischemic stroke	6/226 (2.7)	118/3585 (3.3)
Total	64/226 (28.3)	470/3585 (13.1)

Denominators represent incomplete data points.
*Of the patients with a cardiac thrombus: 1 patient with myocardial infarction died of ischemic stroke.
†Of the patients without a cardiac thrombus: 2 patients with ischemic stroke died due to a cardiac cause, 1 patient with myocardial infarction died of ischemic/hemorrhagic stroke, 2 patients with ischemic stroke had a myocardial infarction.

Table S11. Outcomes at 90-day follow-up including hospital location as random effect

	Cardiac thrombus (n=243)	No cardiac thrombus (n=3676)	Adjusted OR	Adjusted OR including hospital as random effect
**mRS***	3 (2-6)	2 (1-4)	1.6 (1.2-2.0)	1.5 (1.2-2.0)
**Mortality**	76/230 (33)	511/3515 (15)	1.6 (1.1-2.3)	1.6 (1.1-2.3)
**Recurrent ischemic stroke**	12/224 (5)	150/3523 (4)	1.4 (0.7-2.5)	1.4 (0.7-2.6)
**MACE**	64/226 (28)	470/3585 (13)	1.4 (1.0-2.0)	1.5 (1.0-2.1)

mRS=Modified Rankin Scale, MACE=Major Adverse Cardiovascular Events, OR=Odds Ratio.
Missing data, n (%): *174 (4): cardiac thrombus 13, no cardiac thrombus 161.
We adjusted for the following potential confounders: 1) functional outcome and mortality: age, sex, history of AF, ischemic heart disease (IHD) and chronic heart failure (CHF), anticoagulant use, pre-stroke mRS, NIHSS, LVO, and intravenous thrombolysis (IVT) treatment and hospital location (random effect), 2) recurrent ischemic stroke: age, sex, history of AF and ischemic stroke or TIA, and anticoagulant use and hospital location (random effect), 3) MACE: age, sex, history of AF, ischemic stroke or TIA, IHD and CHF, anticoagulation use, NIHSS and hospital location (random effect).

Table S12. Atrial fibrillation detected per phase

	**History of AF**	**AF detected on ECG during hospitalization**	**AF detected on rhythm monitoring during hospitalization**	**AF de novo during 90-day follow-up**	**Total AF detected**	**AF never detected**
**Cardiac thrombus (n=243)**	136 (56%)	49 (20%)	17 (7%)	4 (2%)	189 (78%)	54 (22%)
**No cardiac thrombus (n=3676)**	775 (21%)	325 (9%)	143 (4%)	63 (2%)	1172 (32%)	2504 (68%)

AF=Atrial Fibrillation, ECG=Electrocardiogram.

Table S13. Anticoagulation use in patients with a cardiac thrombus detected on cardiac CT

	Anticoagulation use	Reasons for not initiating anticoagulation
**Patients without history of AF and no AF during follow-up (n=54)**	31 (57%) started on anticoagulation	23 (43%) no anticoagulation due to:<br>- in-hospital death (n=10)<br>- thrombus discovery only during research assessment (n=5)<br>- no thrombus detected on other diagnostic modalities (n=2)<br>- hemorrhagic transformation or bleeding risk (n=2)<br>- other reasons (n=4).
**Patients with history of AF and no anticoagulation at baseline (n=61)**	35 (57%) (re)started on anticoagulation<br>19 (re)started within 4 days	26 (43%) no anticoagulation due to:<br>- in-hospital death (n=21)<br>- thrombus discovery only during research assessment (n=2)<br>- hemorrhagic transformation or bleeding risk (n=2)<br>- other reasons (n=1).
**AF detected after cardiac CT (n=4)**	3 (75%) started on anticoagulation	1 (25%) no anticoagulation due to:<br>- death (n=1)

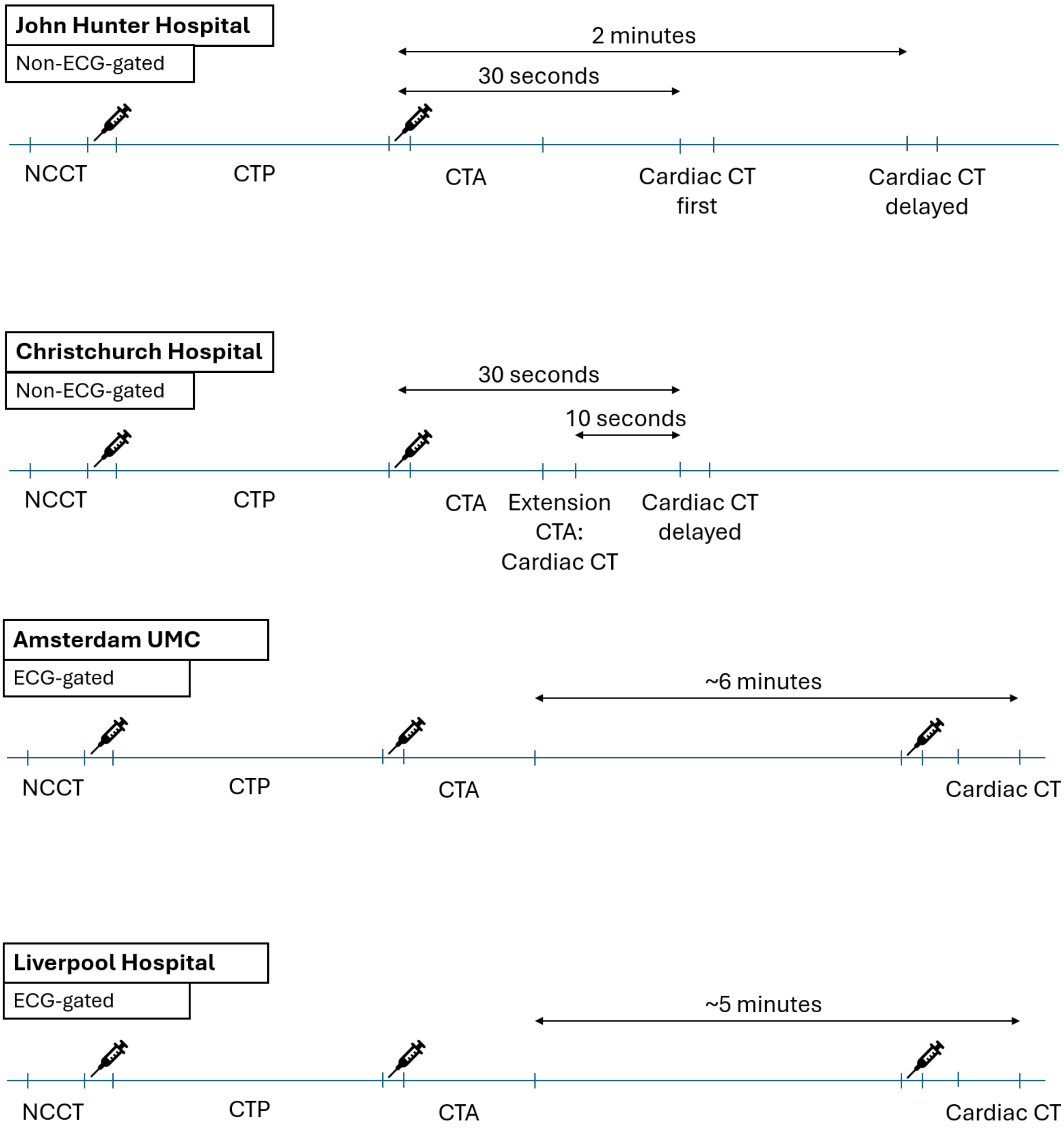

Figure S1. Schematic representation of non-ECG-gated and ECG-gated scan protocols per center

ECG=Electrocardiogram, NCCT=Non-Contrast Computed Tomography, CTP= Computed Tomography Perfusion, CTA=Computed Tomography Angiography. Syringe symbol indicates the administration of a contrast bolus.

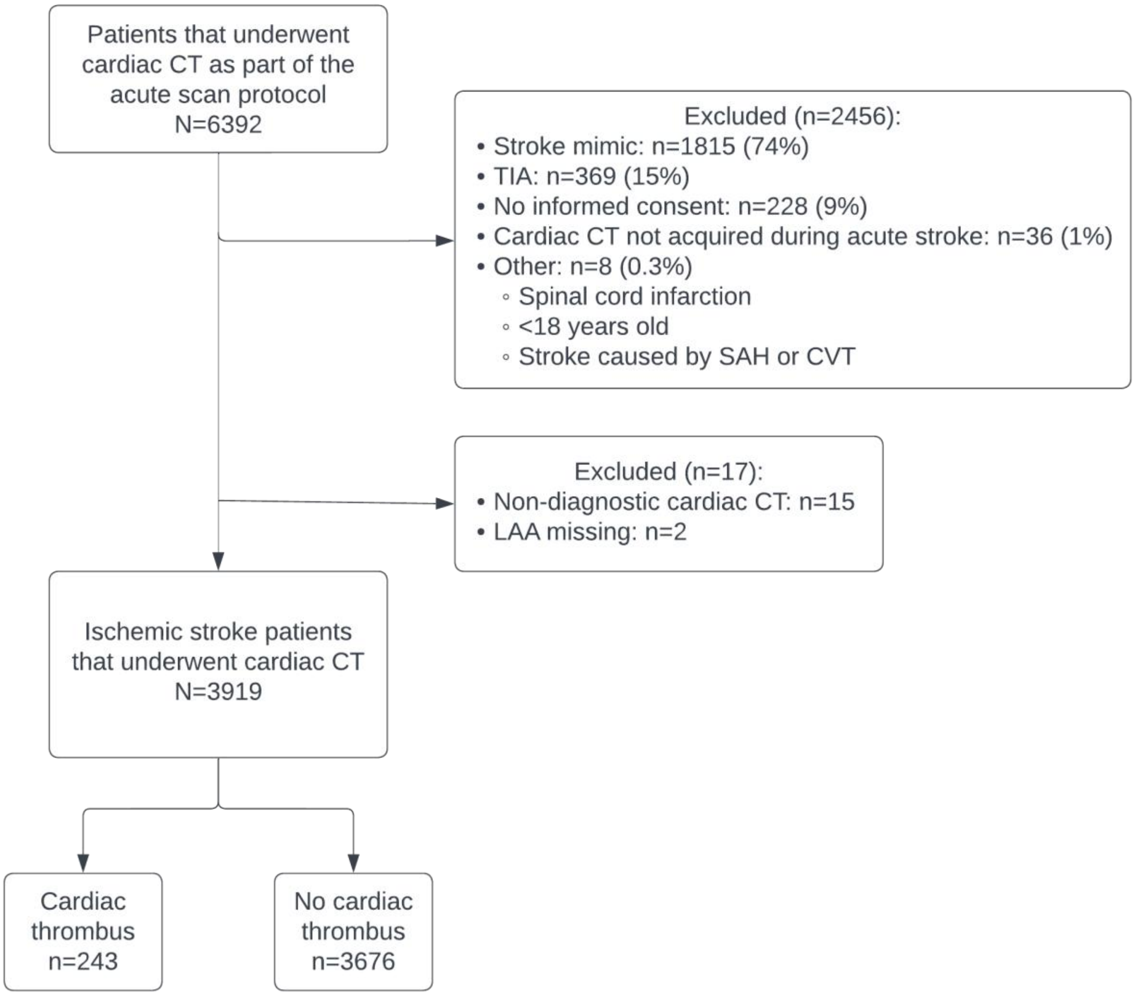

Figure S2. Flowchart

CT=Computed Tomography, TIA=Transient Ischemic Attack, SAH=Subarachnoid Hemorrhage, CVT=Cerebral Venous Thrombosis, LAA=Left Atrial Appendage.

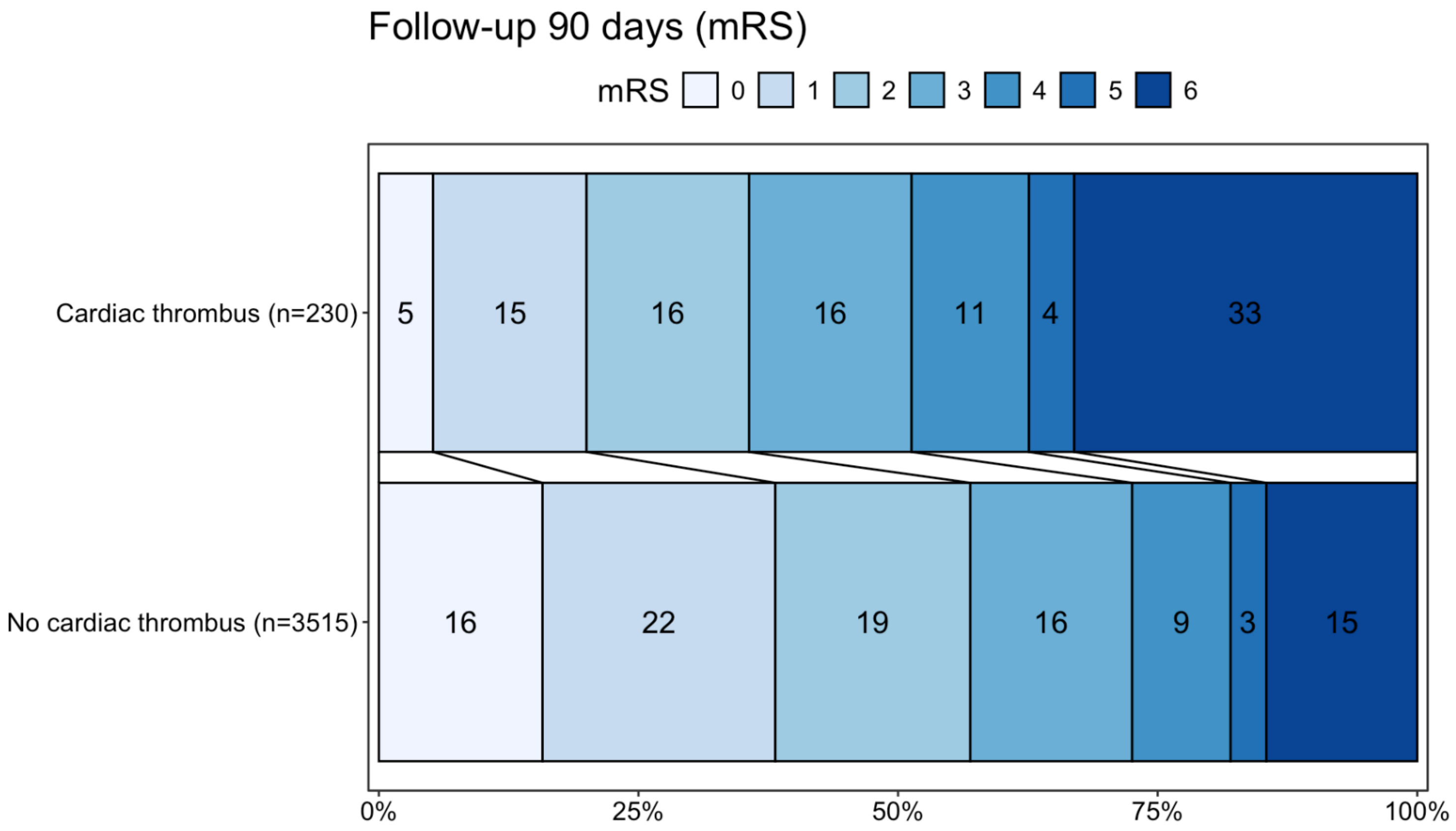

Figure S3. Modified Rankin Scale (mRS)-scores at 90 days
Missing data: cardiac thrombus 13 (5), no cardiac thrombus 161 (4).
